# Effects of varying duty cycle and pulse width on high-intensity focused ultrasound (HIFU)-induced transcranial thrombolysis

**DOI:** 10.1186/2050-5736-1-18

**Published:** 2013-10-01

**Authors:** Thilo Hölscher, Rema Raman, David J Fisher, Golnaz Ahadi, Eyal Zadicario, Arne Voie

**Affiliations:** 1 Brain Ultrasound Research Laboratory (BURL), Department of Radiology, University of California San Diego, San Diego, CA, 92103, USA; 2 Division of Biostatistics and Bioinformatics, University of California San Diego, San Diego, CA, 92093, USA; 3 InSightec, Ltd, 5 Nahum Heth St., Tirat Carmel, 39120, Israel; 4 Department of Neurosciences, University of California San Diego, 200 West Arbor Drive, San Diego, CA, 92103-8756, USA

**Keywords:** Transcranial, High-intensity focused ultrasound, Thrombolysis, Stroke, Pulse width, Duty cycle

## Abstract

The goal was to test the effects of various combinations of pulse widths (PW) and duty cycles (DC) on high-intensity focused ultrasound (HIFU)-induced sonothrombolysis efficacy using an *in vitro* flow model. An ExAblate™ 4000 HIFU headsystem (InSightec, Inc., Israel) was used. Artificial blood clots were placed into test tubes inside a human calvarium and exposed to pulsatile flow. Four different duty cycles were tested against four different pulse widths. For all study groups, an increase in thrombolysis efficacy could be seen in association with increasing DC and/or PW (*p* < 0.0001). Using transcranial HIFU, significant thrombolysis can be achieved within seconds and without the use of lytic drugs *in vitro*. Longer duty cycles in combination with longer pulse widths seem to have the highest potential to optimize clot lysis efficacy.

## Background

Reversible disaggregation of fibrin fibers, improved distribution of plasminogen, tissue plasminogen activator (tPA) within a blood clot, and cavitational mechanisms have been described to explain why ultrasound enhances clot lysis [1-5]. Early clinical studies on transcranial sonothrombolysis in stroke patients using diagnostic ultrasound devices were promising. The most relevant work, so far, was published in 2004 by Alexandrov et al. [6]. Other studies showed comparable results [7-12], suggesting that the use of transcranial diagnostic ultrasound in combination with tPA shortens the time to recanalize vessels significantly. Despite the excitement for this new treatment approach, it is limited to a minority of patients since less than 3% of all stroke victims currently receive tPA. The successful use of HIFU for sonothrombolysis outside the cranium and without the additional use of lytic agents, such as tPA, has been described earlier [13]. Very recent data showing the feasibility of HIFU for transcranial sonothrombolysis in the absence of tPA [14] demonstrate its future potential as a noninvasive, drug-independent treatment option for stroke. However, the data describing the transcranial application for potential use in stroke patients are sparse. Regarding the various ultrasound operating parameters, such as transmit frequency, acoustic output power, duty cycle, and pulse width, it is unclear which combination of these parameters might be preferable to gain efficacious sonothrombolysis in a safe manner, trying to avoid uncontrolled thermal as well as cavitational effects which are most feared in this regard. Hence, thorough research has to be done to define the most efficacious combinations of ultrasound operating parameters to be applied in such a fashion that thermal as well as cavitation effects can be either avoided or, at least, controlled. To date, the knowledge about sonothrombolysis parameter optimization is very limited. One of the most relevant works in this regard was published by Schafer et al. [15]. They tested the effect of operating parameters on the thrombolytic potency of ultrasound and found that thrombolytic efficiency of ultrasound depends directly on duration, intensity, duty cycle, and pulse length, and inversely on frequency. It is important to mention, however, that the knowledge gained by Schafer et al. is based on transmit frequencies between 2.0 and 4.5 MHz. The transmit frequency of the device used for the present study is 220 kHz. Therefore, it is questionable whether the results can be translated to low-frequency ultrasound systems. The goal of this study was to investigate the impact of varying duty cycles and pulse width on HIFU-induced thrombolysis efficacy, in the absence of tPA, in a transcranial *in vitro* flow model.

## Methods

### Ultrasound system and operating parameters

**Figure 1:**
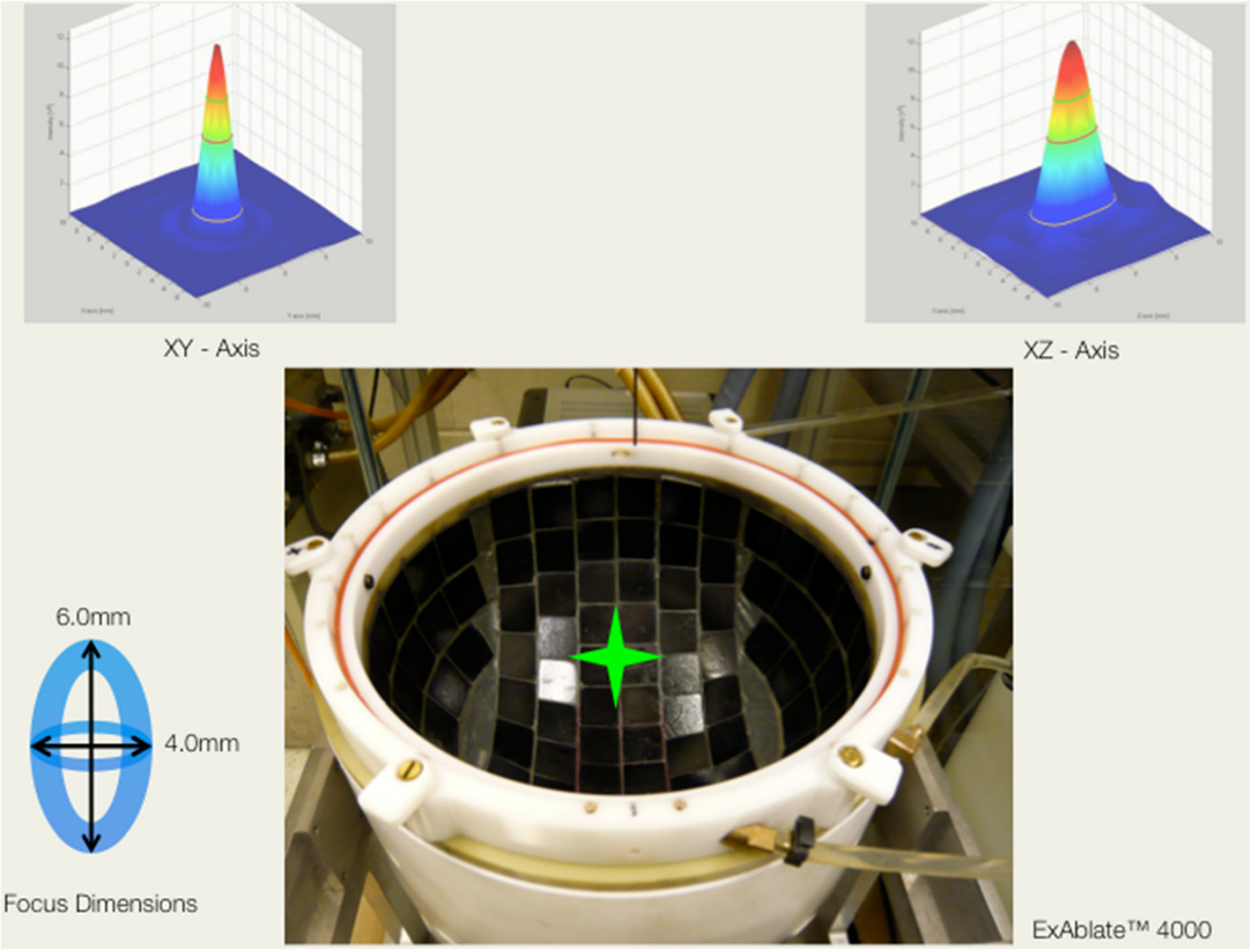
**Top view inside the hemispheric transducer of the ExAblate™ 4000 HIFU headsystem.** Each black tile contains nine single ultrasound-transmitting elements (total *N* = 1,000). All elements transmit towards the center of the transducer (green star), creating a sharp focus beam of 4.0-mm diameter in lateral and 6.0 mm in elevational orientation. Parametric images of the focus, based on acoustic measurements, are given in *XY* (upper left) and *Z* (upper right) orientations.

The ExAblate™ 4000 (InSightec Inc., Tirat Carmel, Israel) HIFU headsystem has been developed for brain applications. A key component of this system is a hemispheric phased array transducer with 1,000 single piezo elements that can be operated independently. Due to its geometry, a sharp focus can be generated, located in the center of the transducer (Figure 1). The transmit frequency of the system is 220 kHz.

To test the impact of various duty cycles and pulse widths on thrombolysis, the decision was made to retain an insonation duration of 30 s and an acoustic output power of 235 W for all studies. The parameters above were chosen due to own data in [14], which show that transcranial thrombolysis can be achieved using these parameters. In the present study, four different duty cycles were tested in combination with four different pulse widths, as displayed in Table 1.

**Table 1:** **Combinations of DC, PW, and the total *N *** **per study group**

**DC (%)**	**PW**			
	**0.1 ms**	**1 ms**	**10 ms**	**100 ms**
5	*N* = 40	*N* = 40	*N* = 40	*N* = 40
10	*N* = 40	*N* = 44	*N* = 40	*N* = 40
20	*N* = 41	*N* = 40	*N* = 41	*N* = 43
50	*N* = 43	*N* = 40	*N* = 42	*N* = 44

*DC*, duty cycles; *PW*, pulse width.

### Clot preparation

Venous whole blood was drawn from healthy, unmedicated donors and transferred into citrate tubes. The donors were enrolled after signing a written informed consent according to the local Institutional Review Board approval (Internal Review Board of the University of California, San Diego). For each clot, 0.5 ml citrate blood was mixed with 40 μl CaCl_2_ (210 mmol/l) and transferred into a borosilicate glass tube, which had a silk thread inside. The clots were incubated for 3 h in a 37°C water bath. The thrombus was organized around the silk thread so that it will be kept in place inside the test tube during insonation. The thrombi had an average weight of 0.2519 g ± 7%. The average length of each thrombus was about 2.5 cm.

### Experimental setup

**Figure 2:**
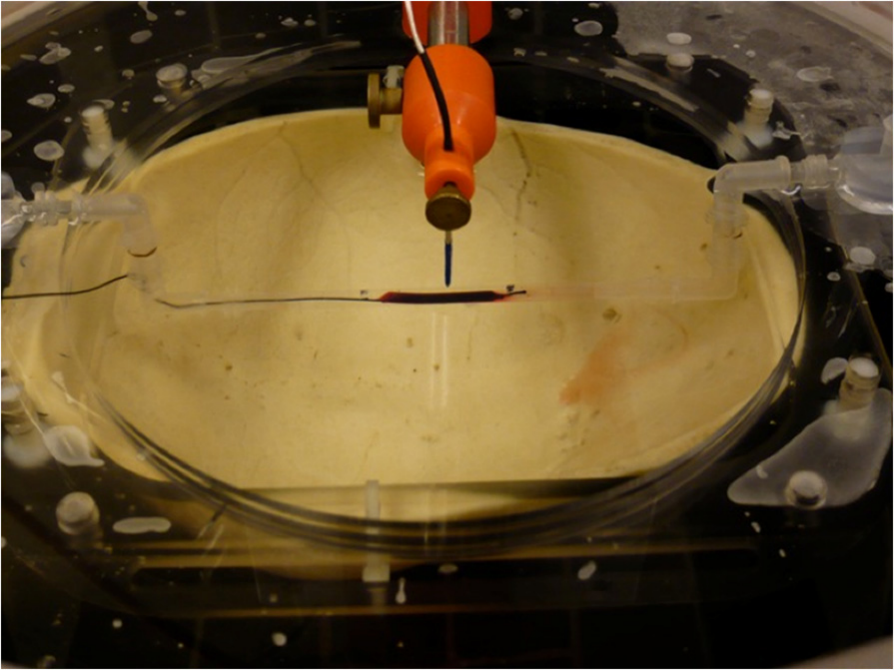
**The hemispheric transducer with the calvarium (upside down) and thrombus inside the test tube.** The tip of the hydrophone is located 2.0 mm above the natural focus position (*x*/*y*/*z* is 0/0/150 mm). The thrombus is centered below the hydrophone tip.

A degassed, cadaveric skull specimen was mounted on an acrylic frame and placed upside down into the degassed water-filled cavity of the hemispheric transducer. The thrombus inside the test tube was placed at the natural focus of the transducer, which is located at the geometrical center in *X*/*Y* and 15 cm from the bottom in the *Z* orientation of the hemispheric array (Figure 2). For all experiments, the clots were exposed to circulating, pulsatile flow (10 ml/min). Phosphate buffered saline was used as a fluid medium. The ambient fluid temperature inside the hemispheric transducer as well as inside the test tube was kept at 24°C.

### Acoustic field mapping

For each of the 16 different duty cycle (DC)/pulse width (PW) combinations, the acoustic parameters spatial peak temporal average intensity (*I*_SPTA_), peak negative pressure (*P*_NEG_), and peak positive pressure (*P*_POS_) were measured using a HIFU hydrophone (Model Y120, Sonic Concepts, Seattle, WA), calibrated for the frequency of 220 kHz. The acoustic measurements are presented in Table 2.

**Table 2:** ***I***_**SPTA**_**, *P***_**NEG**_**, and *P***_**POS **_**pressure values for all 16 parameter combinations**

**Study group**	***I***_**SPTA**_	***P***_**NEG**_	***P***_**POS**_	***N *** **per group**
	**(W/cm**^**2**^**)**	**(MPa)**	**(MPa)**	
1	13.7	2.8	3.1	40
2	13.7	2.8	3.1	40
3	13.7	2.8	3.1	43
4	13.7	2.8	3.1	43
5	27.4	2.8	3.1	43
6	27.4	2.8	3.1	43
7	27.4	2.8	3.1	43
8	27.4	2.8	3.1	43
9	54.7	2.8	3.1	43
10	54.7	2.8	3.1	43
11	54.7	2.8	3.1	43
12	54.7	2.8	3.1	43
13	136.8	2.8	3.1	43
14	136.8	2.8	3.1	43
15	136.8	2.8	3.1	43
16	136.8	2.8	3.1	44

### Statistical analysis

Percent weight loss within each of the 16 DC/PW combinations was compared using a Wilcoxon signed-rank test. A Kruskal-Wallis test was used to examine the differences in clot weight loss among the four PW settings within each DC group. If the overall difference was statistically significant, pair-wise comparisons were performed using a Wilcoxon rank-sum test with the *p* values adjusted using a Bonferroni-Holm correction for multiple comparisons.

## Results

Overall, a total of *N* = 658 experiments were performed. The results are given in Table 3. For each study group, the values for average percent weight loss, standard deviation, and percent weight loss per kilo Joule are given. Three main observations could be made: (1) For all study groups, the percent weight loss was statistically significant (*p* < 0.001). (2) With increasing DCs as well as increasing PWs, the percent weight loss increased as well. (3) At a DC of 50%, the change of PW did not lead to a significant change in weight loss.

**Table 3:** % WT loss mean, Std dev, and pulse ‘off time’ are given for all 16 parameter combinations

	**DC (%)**	**PW**			
		**0.1 ms**	**1 ms**	**10 ms**	**100 ms**
	5	*GP 1*	*GP 2*	*GP 3*	*GP 4*
*N*		40	40	40	40
% Wt loss (mean)		10.28	17.55	23.62	27.15
Std dev		11.95	7.08	11.97	20.75
Off time (ms)		1.9	19	190	1900
	10	*GP 5*	*GP 6*	*GP 7*	*GP 8*
*N*		40	44	40	40
% Wt loss (mean)		17.12	24.32	29.08	42.92
Std dev		9.1	15.88	17.33	24.67
Off time (ms)		0.9	9	90	900
	20	*GP 9*	*GP 10*	*GP 11*	*GP 12*
*N*		41	40	41	43
% Wt loss (mean)		30.02	37.7	39.9	59.6
Std dev		20.12	21.83	21.5	19.16
Off time (ms)		0.4	4	40	400
	50	*GP 13*	*GP 14*	*GP 15*	*GP 16*
*N*		43	40	42	44
% Wt loss (mean)		48.67	53.58	51.43	59.23
Std dev		23.7	20.85	21.85	17.1
Off time (ms)		0.2	2	20	200

The italicized entries represent the 16 different study groups, i.e., GP14 is study group #14.% WT loss, percent weight loss; Std dev, standard deviation.

**Figure 3:**
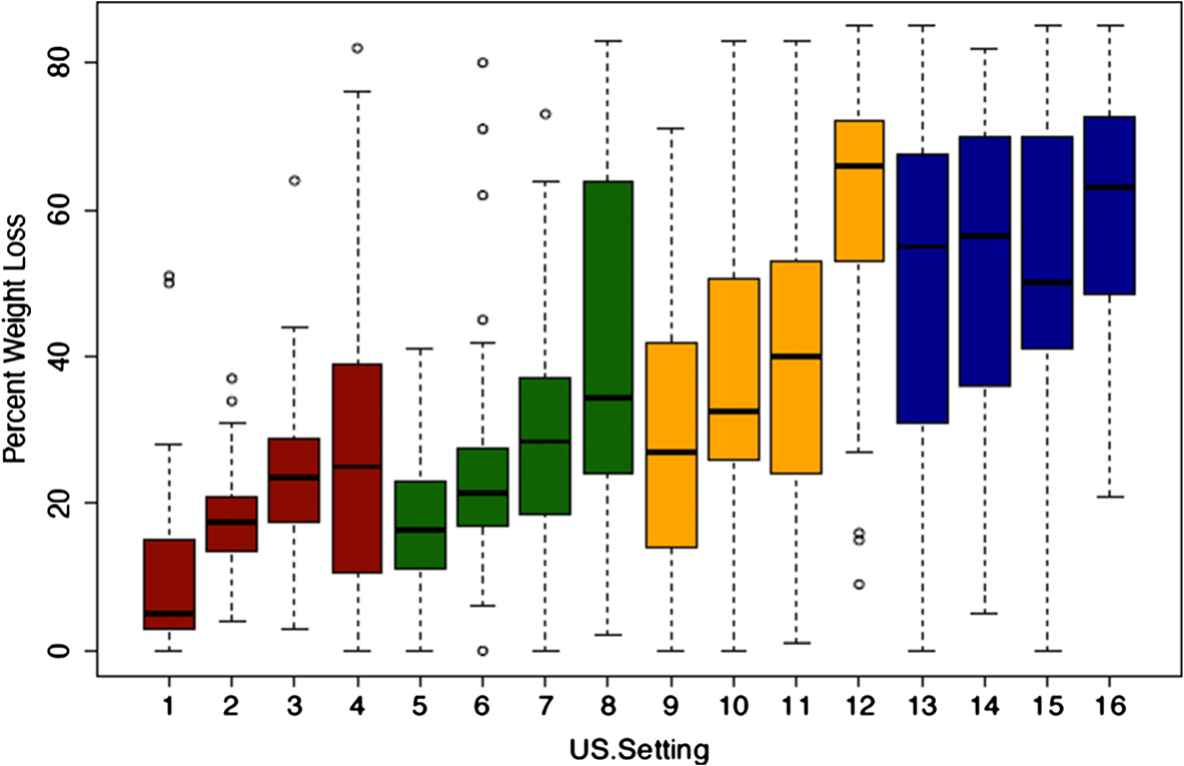
**Histogram showing the percent clot weight loss for all 16 study groups.** 5% DC (red bars), 10% DC (green bars), 20% DC (yellow bars), and 50% DC (blue bars).

A visual presentation of the percent weight loss for each group, displayed as boxplots, is given in Figure 3.

## Discussion

Disregarding the acoustic output power or insonation duration, it could be demonstrated *in vitro* that sonothrombolysis efficacy using transcranial high-intensity focused ultrasound is dependent on the combination of duty cycle and pulse width. Looking at clot weight loss over time, increased duty cycles in combination with increased pulse widths seem to be preferable.

The observation of higher clot weight loss with increased duty cycles and pulse widths might be rather obvious at first sight. As shown in Table 2, the *I*_SPTA_ increases with higher duty cycles, whereas the *I*_SPTA_ for a 5% duty cycle is 13.7 W/cm^2^; in combination with any given pulse width, it increases to tenfold using a duty cycle of 50% (136.8 W/cm^2^). Accordingly, the energy deposition at the clot site increases from 100 to 1,000 J. This observation is in accordance, to some extent, with the work published by Schafer et al. [15]. Using a nonfocused, diagnostic ultrasound transducer in an *in vitro* sonothrombolysis setup, three different duty cycles were tested in combination with increasing pulse widths between 100 and 400 μs. With regard to the parameter duty cycle, the highest amount of thrombolysis efficacy was seen using a 100% duty cycle, i.e., continuous wave mode. The smallest amount of clot lysis was seen using a 20% duty cycle. With regard to the operating parameter pulse width, the investigators described only a modest effect. Given a constant duty cycle, increased pulse widths caused higher clot weight loss as well, however, to less extent. This could be confirmed in the present study. A possible reason why an increase in clot weight loss can be observed with greater pulse widths, although duty cycle, intensity, and total energy remain constant, might be that the total number of pulses per insonation duration decreases. That implies that each pulse simply contains more energy and, therefore, presumably improves clot/fibrin disaggregation. Noteworthy is the observation that increased clot weight loss was rather distinct using short duty cycles in combination with increased pulse widths, whereas the change of pulse width using longer duty cycles, such as 50% in the present study, diminished.

In the present study, only the parameters duty cycle and pulse length were changed, whereas the parameters acoustic output power and insonation duration remained unchanged. Accordingly, the acoustic intensities varied between the study groups with the lowest *I*_SPTA_ seen in the 5% DC and the highest *I*_SPTA_ values in the 50% duty cycle groups, as shown in Table 2. Rosenschein et al. [13] followed a similar approach to investigate the potential impact of duty cycle and pulse length on clot lysis efficacy. They tested five pulse lengths (50, 100, 200, 300, and 400 μs) against various duty cycles (10%, 5%, 4%, 3.3%, and 2.5%), but—different from the present study—they adapted the acoustic output power to keep the *I*_SPTA_ at the focal spot constant at 40 W/cm^2^ throughout all study groups. The total insonation duration was 4 min and remained unchanged as well. Differing from the present study, they found an optimized clot lysis efficiency using a short duty cycle of 4% and a pulse length of 200 μs.

The major limitation of the present study is the missing translation into an appropriate *in vivo* model. A sonothrombolysis rabbit model has been established recently to investigate whether the rather promising *in vitro* results can be confirmed in a living system. Of greatest importance will be to investigate whether this application might cause vascular damage or unwanted blood–brain barrier opening, and if the latter should be the case, what could be done to significantly improve the safety profile. In addition, temperature assessments are missing in the present work. Although there is a common belief that the underlying mechanism of clot/fibrin disaggregation is of mechanical nature, thermal effects cannot be excluded until appropriate measurements or appropriate calculations are available, more so since temperature elevation during sonothrombolysis would be a serious safety issue. Regarding safety, potential clot fragmentation is of concern as well.

## Conclusion

Using transcranial HIFU, significant thrombolysis can be achieved within seconds and without the use of lytic drugs. Aiming ultrasound operating parameter optimization, the present data show that the combination of long duty cycles and long pulse widths seems preferable. A duty cycle of 50% seems to represent an upper limit above which a change of the pulse width does not affect the thrombolysis efficacy. The current findings might be taken into consideration for future translational research on HIFU-induced thrombolysis. The development of a noninvasive, transcranial ultrasound technology which provides the possibility to treat stroke victims who are not eligible for tPA would be a significant achievement in today's stroke care.

## Competing interests

The authors declare that they have no competing interests.

## Authors’ contributions

TH designed the study and the experimental setup, supervised all aspects of the project, and wrote the manuscript. RR did the statistical work-up of the data. DJF was involved in the experimental setup and supervised and participated in the data collection. GA acquired most of the data and prepared them for statistical analysis. EZ participated in the planning of the experiment and provided technical support. AV performed all acoustic measurements and contributed to the experimental design and manuscript revisions. All authors read and approved the final manuscript.

## References

[B1] Braaten JV Goss RA Francis CW Ultrasound reversibly disaggregates fibrin fibers Thromb Haemost 1997 78 3 1063 8 9308755

[B2] Devcic-Kuhar B Pfaffenberger S Gherardini L Mayer C Groschl M Kaun C Benes E Tschachler E Huber K Maurer G Wojta J Gottsauner-Wolf M Ultrasound affects distribution of plasminogen and tissue-type plasminogen activator in whole blood clots in vitro Thromb Haemost 2004 92 5 980 5 15543323 10.1160/TH04-02-0119

[B3] Datta S Ammi AY Coussios CC Holland CK Monitoring and simulating stable cavitation during ultrasound-enhanced thrombolysis J Acoust Soc Am 2007 122 3052

[B4] Datta S Coussios CC McAdory LE Tan J Porter T De Courten-Myers G Holland CK Correlation of cavitation with ultrasound enhancement of thrombolysis Ultrasound Med Biol 2006 32 8 1257 67 10.1016/j.ultrasmedbio.2006.04.008 16875959 PMC1937506

[B5] Prokop AF Soltani A Roy RA Cavitational mechanisms in ultrasound-accelerated fibrinolysis Ultrasound Med Biol 2007 33 6 924 33 10.1016/j.ultrasmedbio.2006.11.022 17434661

[B6] Alexandrov AV Molina CA Grotta JC Garami Z Ford SR Alvarez-Sabin J Montaner J Saqqur M Demchuk AM Moyé LA Hill MD Wojner AW CLOTBUST Investigators Ultrasound-enhanced systemic thrombolysis for acute ischemic stroke N Engl J Med 2004 351 21 2170 8 10.1056/NEJMoa041175 15548777

[B7] Alexandrov AV Mikulik R Ribo M Sharma VK Lao AY Tsivgoulis G Sugg RM Barreto A Sierzenski P Malkoff MD Grotta JC A pilot randomized clinical safety study of sonothrombolysis augmentation with ultrasound-activated perflutren-lipid microspheres for acute ischemic stroke Stroke 2008 39 5 1464 9 10.1161/STROKEAHA.107.505727 18356546 PMC2707058

[B8] Eggers J Seidel G Koch B Konig IR Sonothrombolysis in acute ischemic stroke for patients ineligible for rt-PA Neurology 2005 64 6 1052 4 10.1212/01.WNL.0000154599.45969.D6 15781825

[B9] Eggers J Konig IR Koch B Handler G Seidel G Sonothrombolysis with transcranial color-coded sonography and recombinant tissue-type plasminogen activator in acute middle cerebral artery main stem occlusion: results from a randomized study Stroke 2008 39 5 1470 5 10.1161/STROKEAHA.107.503870 18340100

[B10] Dinia L Rubiera M Ribo M Maisterra O Ortega G del Sette M Alvarez-Sabin J Molina CA Reperfusion after stroke sonothrombolysis with microbubbles may predict intracranial bleeding Neurology 2009 73 10 775 80 10.1212/WNL.0b013e3181b6bb45 19738172

[B11] Molina CA Barreto AD Tsivgoulis G Sierzenski P Malkoff MD Rubiera M Gonzales N Mikulik R Pate G Ostrem J Singleton W Manvelian G Unger EC Grotta JC Schellinger PD Alexandrov AV Transcranial ultrasound in clinical sonothrombolysis (TUCSON) trial Ann Neurol 2009 66 1 28 38 10.1002/ana.21723 19670432

[B12] Molina CA Ribo M Rubiera M Montaner J Santamarina E Delgado-Mederos R Arenillas JF Huertas R Purroy F Delgado P Alvarez-Sabín J Microbubble administration accelerates clot lysis during continuous 2-MHz ultrasound monitoring in stroke patients treated with intravenous tissue plasminogen activator Stroke 2006 37 2 425 9 10.1161/01.STR.0000199064.94588.39 16373632

[B13] Rosenschein U Furman V Kerner E Fabian I Bernheim J Eshel Y Ultrasound imaging-guided noninvasive ultrasound thrombolysis: preclinical results Circulation 2000 102 2 238 45 10.1161/01.CIR.102.2.238 10889137

[B14] Holscher T Fisher D Raman R Ernstrom K Zadicario E Bradley W Voie A Noninvasive transcranial clot lysis using high intensity focused ultrasound J Neurol Neurophysiol 2011 10.4172/2155-9562.S1-002

[B15] Schafer S Kliner S Klinghammer L Kaarmann H Lucic I Nixdorff U Rosenschein U Daniel WG Flachskampf FA Influence of ultrasound operating parameters on ultrasound-induced thrombolysis in vitro Ultrasound Med Biol 2005 31 6 841 7 10.1016/j.ultrasmedbio.2005.03.005 15936499

